# Effect of Creatinine on Various Clinical Outcomes in Patients with Severe Traumatic Brain Injury (TBI)

**DOI:** 10.3390/metabo15100657

**Published:** 2025-10-04

**Authors:** Sarah Dawson-Moroz, Schneider Rancy, George Agriantonis, Kate Twelker, Navin D. Bhatia, Zahra Shafaee, Jennifer Whittington, Bharti Sharma

**Affiliations:** 1 Department of Surgery, East Tennessee State University, Johnson City, TN 37614, USA; morozs@mail.etsu.edu; 2 Department of Surgery, NYC Health and Hospitals—Elmhurst, New York, NY 11373, USA; schneider.rancy@mountsinai.org (S.R.); agriantg@nychhc.org (G.A.); bhatian1@nychhc.org (N.D.B.); shafaeez1@nychhc.org (Z.S.); harrisj20@nychhc.org (J.W.); 3 Department of Surgery, Icahn School of Medicine at Mount Sinai, New York, NY 10029, USA

**Keywords:** creatinine, traumatic brain injury, clinical outcomes, demographics, injury severity, length of stay

## Abstract

**Background:** Traumatic brain injury (TBI) is a major public health concern. Creatinine (Cr) has been well studied as a marker of renal function, specifically the development of acute kidney injury (AKI) in TBI patients. We aimed to evaluate the effect of Cr on various clinical outcomes in patients with severe TBI. **Methods:** We investigated the relationship between Cr levels at various time points and a range of clinical variables, using parametric and non-parametric statistical testing. **Results:** 1000 patients were included in our study. We found a significant association between sex and Cr level at intensive care unit (ICU) admission and ICU discharge. Cr was positively correlated with ISS at hospital admission, ICU admission, ICU discharge, and at death. Conversely, Cr was negatively correlated with GCS at hospital admission, ICU admission, ICU discharge, and at death. Larger decreases in Cr from Hospital to ICU admission were significantly correlated with increased vent days. Larger decreases in Cr from ICU admission to ICU discharge were significantly correlated with increased hospital length of stay (LOS), ICU LOS, and vent days, likely reflecting the degree of initial hypercreatinemia. For all patients, there were significant positive correlations between Cr at admission and ICU LOS, Cr at ICU admission and ICU LOS, and Cr at ICU admission and vent days. **Conclusions:** Our findings support existing literature that demonstrates a positive relationship between Cr levels, ICU LOS, and vent days amongst patients with severe TBI. These data suggest renal injury is predictive of TBI outcomes. Future research should investigate the role of renal therapeutic interventions in TBI recovery.

## 1. Introduction

Creatinine (Cr) is chemically known as α-methyl guanidinoacetic acid, and is the final breakdown product of creatine phosphate in skeletal muscle, produced at a relatively constant rate proportional to muscle mass. Serum creatinine is formed through a spontaneous non-enzymatic anhydration of creatine in muscle cells. Levels are influenced by muscle mass, age, sex, and certain medications. As creatinine is freely filtered by the glomerulus and minimally secreted by renal tubules, it is the most widely used endogenous marker for estimating glomerular filtration rate (GFR) in clinical practice [1]. As such, a rise in creatinine is central to the diagnosis and staging of acute kidney injury (AKI). However, serum creatinine levels may lag behind acute changes in GFR, particularly in the early phase of AKI or after trauma, where rapid shifts in renal function and muscle metabolism occur [2].

In the context of traumatic brain injury (TBI) and major trauma, serum creatinine is often used to diagnose and stage acute kidney injury (AKI). AKI is a frequent and early complication after TBI, occurring in approximately 10–19% of patients, and is independently associated with increased mortality and worse neurological outcomes [3,4,5]. A 2021 study investigating occurrence rate, risk factors, timing, and association with outcome of acute kidney injury in a large cohort of TBI patients across sixty-five ICUs in Europe reported that patients with AKI had a significantly increased ICU length of stay compared to patients without AKI, and that AKI occurrence was associated with increased ICU and overall mortality [3].

Hemodynamic instability, the use of nephrotoxic agents such as mannitol or vancomycin, rhabdomyolysis, and systemic inflammatory responses are all factors that can contribute to AKI and altered creatinine levels in TBI [5]. Additionally, serum creatinine at admission is an independent risk factor for subsequent AKI in TBI patients [5,6]. Some studies have also noted augmented renal clearance following TBI, especially in younger patients, leading to deceptively low serum creatinine despite increased glomerular filtration [7,8]. Thus, close monitoring of renal function and creatinine trends is essential in the management of TBI.

TBI is defined as an alteration in brain function, or other evidence of brain pathology, caused by an external mechanical force such as a blow, jolt, or penetration to the head. TBIs are classified by severity—mild, moderate, or severe—based on clinical criteria such as the Glasgow Coma Scale (GCS), duration of loss of consciousness, and presence of amnesia or focal neurological deficits [9,10,11]. GCS is the most widely used clinical scale to measure the severity of TBI, assessing eye, verbal, and motor responses to categorize TBI as mild (GCS 13–15), moderate (GCS 9–12), or severe (GCS ≤ 8) [12]. TBI severity can also be measured using the Abbreviated Injury Scale (AIS), an anatomical scoring system that uses clinical and imaging findings to rate injury severity in six body regions (head/neck, face, thorax, abdomen, extremities, external) on a scale from 1 (minor) to 6 (maximal/fatal) [13]. The Injury Severity Score (ISS) is a widely used composite score derived from AIS scores. It is calculated by summing the squares of the three highest AIS scores from different body regions. An ISS > 15 is commonly used to define major trauma [14].

Mild TBI, or concussion, accounts for the majority of TBI cases and is characterized by transient neurological dysfunction, typically with GCS 13–15, brief or no loss of consciousness, and no abnormalities on standard neuroimaging. Most patients with mild TBI recover within weeks, though a small percentage may experience persistent symptoms (post-concussive syndrome), including headache, dizziness, cognitive impairment, and mood disturbances [9,15,16]. Moderate and severe TBIs are associated with more pronounced and prolonged impairment, including risk of permanent disability or death. The primary injury, or mechanical disruption of brain tissue at the moment of trauma, is followed by secondary injury, such as delayed cellular and molecular cascades (i.e., inflammation, oxidative stress, and neurovascular dysfunction) [9,17].

Based on the most recent CDC data, there were approximately 214,110 TBI-related hospitalizations in 2020 and 69,473 TBI-related deaths in 2021 [18]. People aged 75 years and older account for about 32% of TBI-related hospitalizations and 28% of TBI-related deaths. Additionally, males are nearly two times more likely to be hospitalized and three times more likely to die from a TBI than females [18]. TBI is a major public health concern, with millions of cases occurring annually worldwide. Management is tailored to severity, with acute stabilization, prevention of secondary injury, and multidisciplinary rehabilitation as key components for moderate-to-severe cases [10,11].

Creatinine is a well-studied endogenous marker of GFR and is frequently used to stage AKI in the setting of trauma. Our study aimed to analyze the relationship of creatinine to several clinical outcomes and timeframes amongst patients admitted with severe TBI.

## 2. Methods

We performed a single-center, retrospective review at Elmhurst Hospital, a Level 1 trauma center in Queens, New York City. All patients who presented to the hospital with a severe traumatic brain injury, defined as an AIS score of 3 or higher, between 1 January 2020 and 31 December 2023, were included in the study. Patients who tested positive for COVID-19 at the time of admission, those who died or were discharged within 24 h of their original injury, and those who had non-severe and minor injuries were excluded. Patient data were obtained from the National Trauma Registry of the American College of Surgeons (NTRACS) database at our institution. Data extracted and organized in Excel included demographics (sex, age, race, ethnicity), injury type (blunt vs. penetrating), mechanism of injury (e.g., fall, motor vehicle collision, assault), diagnosis (e.g., concussion, subdural hematoma, subarachnoid hemorrhage), and number of neurologic injuries (one to four or more). Raw creatinine data was categorized as extreme hypocreatinemia, hypocreatinemia, normal, hypercreatinemia, and extreme hypercreatinemia. After data review, a final cohort of 1000 patients was included in the analysis.

### Statistical Analysis

Descriptive summary statistics were produced for male and female patients with means and standard deviations for continuous variable outcomes and frequencies and percentages for categorical variable outcomes. Differences between male and female patients were assessed using a two-tailed Student’s *t*-test for continuous variables, Pearson’s *X*^2^ test for categorical variables, and Mann–Whitney *U* test for ordinal variables.

Creatinine levels at all five time points were compared against demographic and clinical factors using two-tailed *t*-test for 2-category analyses (sex, injury type) and an ANOVA one-way test with Welch’s transformation for analyses of more than 2-categories (age range, injury mechanism, diagnosis, number of injuries).

The effect of creatinemia category on mean GCS and ICS scores across all time points was investigated with a Kruskal–Wallis test to approximate ranked ANOVA testing. In order to account for unequal population sizes and any unequal variances between groups, correlation between raw creatinine values at all time points and mean GCS and ISS was assessed with Spearman’s rho correlation. Relationships between clinical outcomes and changes in creatinine, as well as creatinine at hospital admission, ICU admission, and ICU discharge, were assessed with univariable and multivariable linear regression analyses.

A *p*-value < 0.05 was considered statistically significant. All analyses were conducted in Stata 19/SE (StataCorp, College Station, TX, USA).

## 3. Results

This study consisted of a cohort of 1000 patients (Table 1; 23% female, N = 232). Most patients (N = 793, 64.26%) received the diagnosis of intraparenchymal hemorrhage (IPH). This was followed by subarachnoid hemorrhage (SAH; N = 424, 34.36%) and concussion (N = 15, 1.22%). Compared to female patients, male patients were significantly younger on average (49 vs. 65 years), were heavier (91 vs. 74 kg), had lower mean GCS (9 vs. 15), greater mean ventilator days (2.13 vs. 0.80 days), and greater ICU LOS (4.11 vs. 2.66 days). For male patients, Cr was statistically greater at ICU admission (0.6 vs. 0.45 mg/dL) and ICU discharge (0.58 vs. 0.37 mg/dL).

When Cr level across hospital admission was investigated for any association between clinical and demographic factors, significant associations between sex and Cr level at intensive care unit (ICU) admission (*p* = 0.001) and ICU discharge (*p* = 0.005) were evident (Table 2). No significant associations were found between other demographic or clinical factors and creatinine levels at any of the measured time points.

Creatinine levels across all timepoints were grouped into predefined categories to assess for differences in average GCS and ISS between groups using a Kruskal–Wallis test to approximate a ranked ANOVA test. Creatinemia category at hospital admission had a significant effect on mean ISS (*p* = 0.001) but not on mean GCS (*p* = 0.149). Similarly, at ICU admission, mean GCS (*p* = 0.001) and ISS (*p* = 0.000) scores were significantly different between creatinemia categories. There was no significant difference in mean GCS and ISS between creatinemia categories at ICU discharge or hospital discharge. At point of death, there was a statistically significant difference between creatinemia groups in mean ISS (*p* = 0.003) and GCS (*p* = 0.039) scores (Table 3).

To account for the possibility of creatinemia categorization causing a type II error due to small group sizes, direct correlations between Cr level at each time point and mean GCS and ISS were investigated using ranked sum Spearman’s correlation test. There was a significant positive correlation between mean ISS and average Cr at hospital admission (*r* = 0.106, *p* = 0.000), Cr at ICU admission (*r* = 0.179, *p* = 0.000), Cr at ICU discharge (*r* = 0.104, *p* = 0.001), and Cr at death (*r* = 0.104, *p* = 0.001). Conversely, there was a statistically significant negative correlation between mean GCS score and average Cr at hospital admission (*r* = −0.114, *p* = 0.001), Cr at ICU admission (*r* = −0.182, *p* = 0.000), Cr at ICU discharge (*r* = −0.103, *p* = 0.001), and Cr at death (*r* = −0.097, *p*= 0.002) (Table 4).

**Figure 1:**
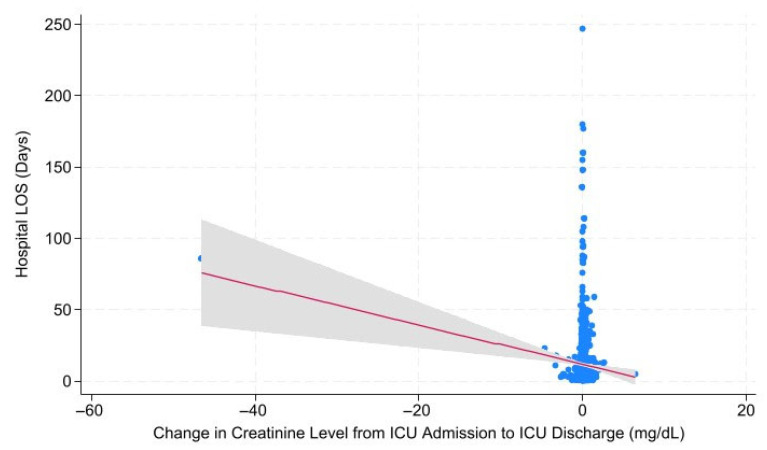
Change in ICU Admission to ICU Discharge: Creatinine vs. Hospital Length of Stay. The relationship between hospital length of stay in days and change in creatinine level from ICU admission to ICU discharge, demonstrating a statistically significant negative correlation (*p* = 0.001). Abbreviations: LOS = Length of Stay; ICU = Intensive Care Unit.

Linear regression modeling was performed to investigate correlations between changes in creatinine level from hospital to ICU admission and from ICU admission to ICU discharge with hospital LOS, ICU LOS, days on mechanical ventilation, and mortality. Change in Cr from Hospital to ICU admission was significantly negatively correlated with ventilator days (*p* = 0.045; *r* = −0.500). Change in Cr from ICU admission to ICU discharge was significantly negatively correlated with hospital LOS (Figure 1; *p* = 0.001; *r* = −1.380), ICU LOS (*p* = 0.000; *r* = −0.542), and ventilator days (*p* = 0.011; *r* = −0.322) (Table 5).

To control for any confounding effects contributing to the significant univariable correlations, a multivariable linear regression model was conducted to test if the significant correlations between changes in Cr level and hospital LOS, ICU LOS, ventilator Days, and mortality remained so when controlling for age, race, ethnicity, and weight. On multivariable analysis, change in Cr level from hospital to ICU admission was no longer found to significantly correlate with ventilator days (*p* = 0.090), however changes in Cr from ICU admission to ICU discharge continued to demonstrate statistically significant correlations with hospital LOS (*p* = 0.001), ICU LOS (*p* = 0.000), and ventilator days (0.014) (Table 6).

**Figure 2:**
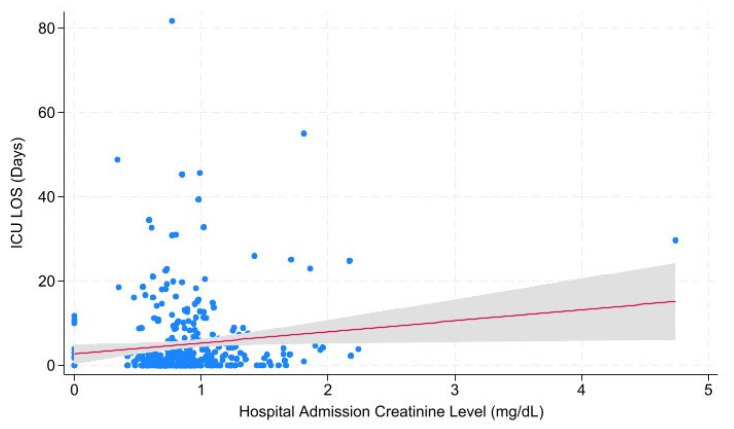
Hospital Admission Creatinine Level vs. ICU Length of Stay for Patients with Two Injuries. The significant relationship between creatinine level at the time of hospital admission and ICU length of stay in days among patients who sustained two injuries. Abbreviations: ICU = Intensive Care Unit; LOS = Length of Stay.

**Figure 3:**
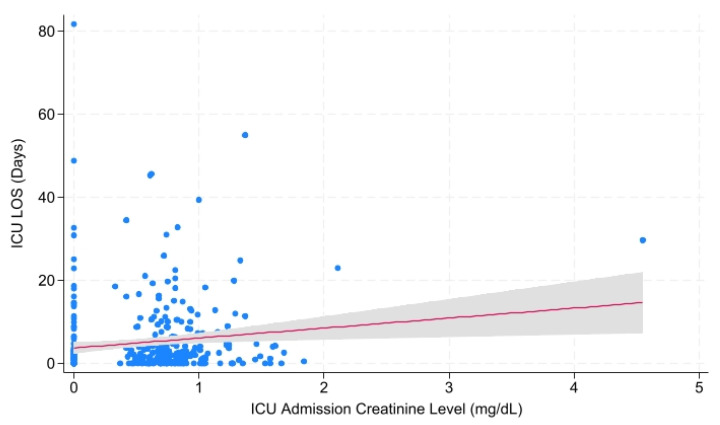
ICU Admission Creatinine Level vs. ICU Length of Stay for Patients with Two Injuries. The significant relationship between creatinine level at the time of ICU admission and ICU length of stay in days among patients who sustained two injuries. Abbreviations: ICU = Intensive Care Unit; LOS = Length of Stay.

Correlations of creatinine level across admission with hospital LOS, ICU LOS, ventilator days, and mortality were investigated with univariable linear regression (Table 7). Overall, there were significant correlations between Cr at admission and ICU length of stay (*p* = 0.043; *r* = 0.586), Cr at ICU Admission and ICU length of stay (*p* = 0.001; *r*= 1.029), and Cr ICU Admission and vent days (*p* = 0.031; *r* = 0.576). For patients with 1 neurologic injury (N = 508), there were significant correlations between Cr at ICU admission and hospital length of stay (*p* = 0.024; *r* = 1.238), ICU length of stay (*p* = 0.003; *r* = 0.766), and vent days (*p* = 0.002; *r* = 0.508). For patients with 2 neurologic injuries (N = 367), there were significant correlations between Cr at admission and ICU length of stay (Figure 2; *p* = 0.029; *r* = 2.629) and between Cr at ICU admission and ICU length of stay (Figure 3; *p* = 0.010; *r* = 2.399).

No significant associations were noted amongst patients who sustained three neurologic injuries or four or more neurologic injuries. However, this subset comprised a relatively small cohort (N = 89 for three injuries and N = 19 for four or more injuries). Thus, these findings may represent a Type II error.

To control for any confounding effects, a similar multivariable linear regression model was conducted to control for sex, age, race, ethnicity, and weight. On multivariable regression analysis, all univariable regressions remained significant, with the exception that Cr at ICU admission was no longer statistically correlated with ventilatory days (*p* = 0.062) (Table 8).

## 4. Discussion

The impact of creatinine levels among patients suffering severe TBI has been well studied, but mostly in the context of AKI, often defined by changes in serum creatinine, and its association with ICU outcomes in TBI populations [3,4,5,6,7,8,19,20]. Large multicenter cohort studies have demonstrated that AKI is associated with increased ICU length of stay and worse neurological outcomes in TBI patients. The Collaborative European NeuroTrauma Effectiveness Research in Traumatic Brain Injury (CENTER-TBI) study found that AKI (using KDIGO [21] creatinine criteria) was associated with longer ICU stays and higher mortality at 6 months [3]. Similarly, severe AKI (stage 3 or greater, based on creatinine) has been linked to increased hospital length of stay and greater need for tracheostomy and gastrostomy, which are indirect markers of prolonged ventilator dependence [19]. While our study did not examine AKI as a composite endpoint in examining TBI outcomes, we did consider the direct relationship between creatinine levels and a number of important clinical outcomes.

When comparing creatinine levels at different time points to demographic factors, we found a significant difference among sexes for creatinine at ICU admission and ICU discharge, with males presenting with higher creatinine levels at these time points. One 2021 study found sex-based differences in serum creatinine response after TBI [22]. In the acute phase, both male and female TBI patients showed changes in creatinine. However, elevated creatinine is associated with improved short-term neurological recovery in males, but not in females [22].

While we did not note any other significant relationships between demographics and creatinine in our study, the existing literature suggests age is a strong predictor of both renal outcomes and overall prognosis after TBI, with patients < 65 years old having a higher risk of developing chronic kidney disease (CKD) post-injury [23,24]. One 2023 case–control study aimed to investigate the clinical outcomes of TBI patients with or without CKD comorbidity at the time of injury and found that ICU length of stay and hospitalization expenses were higher in the CKD group than the non-CKD group, although not statistically significant, and advanced age, low admission GCS score, elevated blood urea, and creatinine levels were significantly associated with a poor neurological prognosis [24].

There is no evidence in the medical literature that injury type (i.e., blunt vs. penetrating), injury mechanism, or specific type of intracranial injury (i.e., subarachnoid hemorrhage, epidural hematoma, etc.) has a significant independent impact on creatinine levels or the risk of AKI in patients with TBI. However, the number of injuries, and specifically whether a patient has isolated TBI or polytrauma, is associated with an increased risk of AKI and higher creatinine levels [3,5,25].

In this study, we did not note significant associations between the number of injuries and measured creatinine at any particular time point. We did, however, find significant relationships between baseline creatinine levels at certain timepoints and specific outcomes such as ICU length of stay and vent days when stratified by number of injuries. Specifically, patients with one or two injuries had a significant relationship between creatinine level at the time of ICU admission and ICU length of stay. This is in keeping with robust research that demonstrates higher creatinine levels and the development of AKI are associated with increased ICU length of stay in patients with TBI [26,27]. Overall, for patients with 1 injury, the creatinine level at ICU admission was significantly associated with ventilator days. As with increased ICU length of stay, previous multicenter studies and meta-analyses have shown that the number of vent days is positively correlated with AKI, as defined by creatinine levels [25,28,29].

Lastly, we found that the range of creatinemia was significantly associated with ISS and GCS scores at ICU admission and patient death timepoints. While the range of creatinemia has not been related to injury severity scoring in previous studies, several existing studies have shown that lower GCS and higher ISS are associated with higher creatinine levels and increased risk of AKI in severe TBI patients [5,20]. Zhang et al. (2025) [5] conducted a systematic review and meta-analysis and reported that lower GCS at admission and GCS ≤ 8 were significantly associated with increased risk of AKI, as defined by elevated creatinine. Higher admission serum creatinine was also identified as a risk factor for AKI in TBI patients. De Cássia et al. (2024) [20] conducted a retrospective cohort study of severe TBI and showed that higher New Injury Severity Score (NISS) and lower GCS were independently associated with both the occurrence and severity of AKI, as well as with an increase in in-hospital mortality.

An important limitation of this study is the wide variability in group sizes evaluated. In addition to a non-normal distribution of data and overlapping variances, this may have contributed to decreased detection of significant differences between groups. Additionally, a large group of patients was categorized as “Other” race, which we could not address due to the retrospective nature of the study. Because this group was so large, it could not be removed for the risk of weakening data analysis. We also lack the assessment of certain demographic variables in our analysis of the relationship between creatinine levels and severe TBI. While age and sex-based differences have been reported in serum creatinine levels following TBI [22,23,24], other demographic factors such as race and ethnicity have also been shown to influence creatinine levels [25]. In addition, pre-existing comorbidities such as diabetes mellitus, hypertension, chronic kidney disease, congestive heart failure, and higher body mass index (BMI) have all been shown to be associated with increased risk of increased creatinine levels and AKI in patients with severe TBI [25,30,31]. Moreover, pre-existing comorbidities such as migraine, mental health conditions, osteoporosis, previous TBI, heart disease, and elevated BMI have been found to have moderate to high predictive value of adverse outcomes following TBI [32]. One recent multicenter retrospective cohort study also found that older age, higher Charleson Comorbidity Index (CCI), GCS scores of 9 or 10, severe trauma, and mechanical ventilation or craniotomy are associated with poor neurological outcome in patients with moderate TBI [33]. Patient data on pre-existing comorbidities was not collected in this study and should be considered as modifying factors in future studies. Secondly, most studies evaluated creatinine in the context of AKI, not as a standalone endpoint. There were few studies on creatinine as it relates to TBI alone, as it is so commonly used to assess GFR and AKI. As a result, the relationship between creatinine and our measured outcomes of interest may have low external validity as it pertains to the measurement of renal function. Finally, our study did not collect data on medications taken at the time of injury. Many drugs, including certain antibacterials, antivirals, cardiovascular agents, and gastrointestinal agents, have been shown to affect serum creatinine levels. Further, nonsteroidal anti-inflammatory drugs (NSAIDs), antivirals such as acyclovir, adefovir, and ganciclovir, cisplatin, and renin-angiotensin system inhibitors are known to induce AKI [34]. As they were not evaluated, we cannot be certain that initial creatinine levels were not affected by outside medications. Creatinine levels can also be affected by the hyperosmolar agents often used to treat TBI, such as mannitol and hypertonic saline [5,35,36]. As these agents can directly impact creatinine levels, the development of AKI, and clinical outcomes, future studies should consider including them in the analysis.

## 5. Conclusions

Our analysis corroborates the existing literature that demonstrates a positive relationship between creatinine levels, ICU length of stay, and ventilator days amongst patients with severe traumatic brain injury. Future research should focus on expanding clinical and demographic factors in the assessment of creatinine changes associated with severe TBI, as well as potentially including measurement of renal function and other clinical endpoints related to creatinine levels.

## Figures and Tables

**Table 1:** Demographics.

	Male	Female	*p*-Value
N (%)	768 (77%)	232 (23%)	
Age (mean ± SD)	48.88 ± 19.87 years	65.04 ± 23.90 years	0.000
Patient Weight (mean ± SD)	91.11 ± 42.09 kg	74.12 ± 36.69 kg	0.000
Creatinine at Various Timepoints			
Hospital Admission (mean ± SD)	0.96 ± 0.80 mg/dL	0.89 ± 0.73 mg/dL	0.241
ICU Admission (mean ± SD)	0.60 ± 0.79 mg/dL	0.45 ± 0.49 mg/dL	0.001
ICU Discharge (mean ± SD)	0.58 ± 1.83 mg/dL	0.37 ± 0.47 mg/dL	0.005
Hospital Discharge (mean ± SD)	0.74 ± 3.19 mg/dL	0.82 ± 3.82 mg/dL	0.770
Death (mean ± SD)	1.48 ± 37.89 mg/dL	0.06 ± 0.34 mg/dL	0.303
Race (*n*, %)			0.000
White	128 (17%)	53 (23%)	
Black	61 (8%)	16 (7%)	
Asian	92 (12%)	55 (23%)	
Native Hawaiian or Other Pacific Islander	2 (0.25%)	2 (1%)	
Other	469 (61%)	102 (44%)	
Unknown	14 (2%)	4 (2%)	
Ethnicity (*n*, %)			0.000
Hispanic	384 (50%)	80 (34%)	
Non-Hispanic	352 (46%)	142 (61%)	
Unknown	31 (4%)	10 (5%)	
Trauma Type (*n*, %)			0.133
Blunt	749 (98%)	230 (99%)	
Penetrating	19 (2%)	2 (1%)	
Glasgow Coma Score (mean ± SD)	9 ± 6	15 ± 0	0.000
Injury Severity Score (mean ± SD)	19.5 ± 1.5	20 ± 2	0.498
Hospital Length of Stay (mean ± SD)	12.32 ± 21.6	9.55 ± 13.72	0.066
ICU Length of Stay (mean ± SD)	4.11 ± 7.63	2.66 ± 5.17	0.007
Ventilator Days (mean ± SD)	2.13 ± 6.88	0.80 ± 2.81	0.004
Injury Pattern (*n*, %)			0.103
EDH	0 (0%)	1 (0%)	
SDH	1 (0%)	0 (0%)	
SAH	338 (28%)	86 (26%)	
IPH	603 (50%)	190 (57%)	
Concussion	11 (1%)	4 (1%)	
Other	248 (21%)	55 (16%)	
Mortality (*n*, %)	83 (11%)	25 (11%)	0.975

Demographic characteristics of the 1000 patients included in the analysis. The cohort was predominantly male (*n* = 768), identified as “other” race (*n* = 571), non-Hispanic (*n* = 494), sustained blunt injuries (*n* = 979), and received a diagnosis of intraparenchymal hemorrhage (*n* = 793). Abbreviations: ICU = Intensive Care Unit; EDH = epidural hematoma; SDH = subdural hematoma; SAH = subarachnoid hemorrhage; IPH = intraparenchymal hemorrhage.

**Table 2:** Comparison of creatinine levels at five time points against demographic and clinical factors.

		Hospital Admission Cr (mg/dL)	ICU Admission Cr (mg/dL)	ICU Discharge Cr (mg/dL)	Hospital Discharge Cr (mg/dL)	Death Cr (mg/dL)
Sex	Female	0.894	0.454	0.373	0.821	0.063
	Male	0.963	0.603	0.577	0.740	1.475
	*p*-value	0.241	0.001	0.005	0.770	0.303
Age Range	Under 18	0.696	0.530	0.434	0.440	0.000
	18–45	0.903	0.552	0.608	0.548	0.077
	46–74	0.973	0.593	0.494	0.800	2.866
	75+	0.974	0.563	0.455	1.08	0.151
	*p*-value	0.283	0.947	0.806	0.443	0.804
Injury Type	Blunt	0.9455	0.569	0.532	0.760	1.168
	Penetrating	0.978	0.519	0.416	0.6901	0.138
	*p*-value	0.754	0.657	0.309	0.617	0.339
Injury Mechanism	Fall	0.981	0.555	0.520	0.880	1.781
	Blunt Assault	0.909	0.634	0.578	0.636	0.071
	MVC	0.886	0.554	0.600	0.464	0.171
	Pedestrian Struck	0.871	0.616	0.530	0.532	0.158
	Micro MVC	0.846	0.564	0.501	0.534	0.026
	Penetrating Assault	0.996	0.545	0.437	0.696	0.145
	Other	0.843	0.302	0.209	0.672	0.000
	*p*-value	0.693	0.802	0.992	0.888	0.997
Diagnosis	SDH	1.550	0.000	0.000	0.000	0.000
	SAH	0.942	0.605	0.628	0.600	0.129
	EDH	0.640	0.460	0.530	0.610	0.000
	IPH	0.962	0.577	0.556	0.809	1.433
	Concussion	1.160	0.786	0.707	0.489	0.293
	Other	0.884	0.560	0.486	0.560	0.097
	*p*-value	>0.05	>0.05	>0.05	>0.05	>0.05
Number of Injuries	One	0.979	0.550	0.457	0.936	2.140
	Two	0.886	0.546	0.595	0.548	0.119
	Three	1.040	0.734	0.700	0.689	0.165
	Four+	0.844	0.732	0.498	0.465	0.104
	*p*-value	0.217	0.124	0.469	0.399	0.831

Cr at five time points (admission, ICU admission, ICU discharge, hospital discharge, and death) against demographics (sex, age range), and clinical factors (injury type, injury mechanism, diagnosis, number of injuries). Males were significantly more likely to have higher creatinine levels than females at ICU admission (*p* = 0.001) and ICU discharge (*p* = 0.005). Abbreviations: Cr = Creatinine; ICU = Intensive Care Unit; MVC = Motor Vehicle Collision; EDH = epidural hematoma; SDH = subdural hematoma; SAH = subarachnoid hemorrhage; IPH = intraparenchymal hemorrhage.

**Table 3:** Effect of Creatinine Category on ISS and GCS Scores Across All Time Points.

		Extreme Hyper-Creatinemia (mg/dL)	Hyper-Creatinemia (mg/dL)	Normo-Creatinemia (mg/dL)	Hypo-Creatinemia (mg/dL)	Extreme Hypo-Creatinemia (mg/dL)	*p*-Value
Cr at Hospital Admission	N	107	61	574	38	179	
	ISS	19.78	21.75	18.17	17.34	18.05	0.011
	GCS	12.05	12.28	12.74	13.32	13.00	0.149
Cr at ICU Admission	N	58	31	321	21	191	
	ISS	20.81	22.16	19.89	18.90	17.29	0.000
	GCS	11.79	12.61	12.16	13.48	13.10	0.001
Cr at ICU Discharge	N	43	11	236	32	259	
	ISS	19.95	17.64	19.21	18.44	18.20	0.069
	GCS	11.84	14.64	12.75	12.53	12.74	0.578
Cr at Hospital Discharge	N	31	17	381	29	255	
	ISS	20.32	19.18	17.83	17.79	18.91	0.461
	GCS	12.26	14.47	13.07	12.86	12.43	0.162
Cr at Death	N	11	2	13	81	11	
	ISS	21.00	26.25	19.10	38.50	18.36	0.003
	GCS	10.33	11.75	11.14	6.50	12.84	0.039

The effect of various levels of creatinemia (extreme hyper-creatinemia, hyper-creatinemia, normo-creatinemia, hypo-creatinemia, and extreme hypo-creatinemia) on ISS and GCS severity rating scores across all five measured time points (hospital admission, ICU admission, ICU discharge, hospital discharge, and death). At ICU admission, the between-group differences were noted for both ISS (*p* = 0.0003) and GCS (*p* = 0.04) scores, with the highest ISS of 22.16 noted for the hyper-creatinemia category and the lowest GCS of 11.79 noted for the extreme hyper-creatinemia category. At the patient death time point, creatinemia category had a significant effect on both mean ISS (*p* = 0.017) and GCS (*p* = 0.001) scores, with the greatest ISS (38.50) and lowest GCS (6.50) being reported for the hypo-creatinemia category Abbreviations: Cr = Creatinine; ICU = Intensive Care Unit; ISS = Injury Severity Score; GCS = Glasgow Coma Scale.

**Table 4:** Correlation of Mean Creatinine at Each Time Point with ISS and GCS.

Timeframe	Mean Cr ± SE (mg/dL)	Scoring System	Rho Coefficient	*p*-Value
Hospital Admission	0.945 ± 0.025	ISS	0.106	0.000
		GCS	−0.114	0.001
ICU Admission	0.568 ± 0.023	ISS	0.179	0.000
		GCS	−0.182	0.000
ICU Discharge	0.529 ± 0.051	ISS	0.104	0.001
		GCS	−0.103	0.001
Hospital Discharge	0.759 ± 0.106	ISS	−0.035	0.279
		GCS	0.005	0.868
Death	1.147 ± 1.051	ISS	0.104	0.001
		GCS	−0.097	0.002

Correlation of creatine as continuous variable with ordinal ISS and GCS severity rating scores across all five measured time points (hospital admission, ICU admission, ICU discharge, hospital discharge, and death). Abbreviations: Cr = Creatinine; ICU = Intensive Care Unit; ISS = Injury Severity Score; GCS = Glasgow Coma Scale.

**Table 5:** Correlation Between Changes in Creatinine Level and Hospital LOS, ICU LOS, Ventilator Days, and Mortality.

Timeframe	Outcome	*p*-Value	Coefficient ± SE [95% CI]
Change in Cr Level from Hospital Admission to ICU Admission	Hospital LOS	0.497	−0.550 ± 0.808 [−2.14–1.04]
	ICU LOS	0.265	−0.321 ± 0.288 [−0.887–0.244]
	Ventilator Days	0.045	−0.500 ± 0.249 [−0.989–−0.011]
	Mortality	0.139	−0.019 ± 0.013 [−0.043–0.006]
Change in Cr Level from ICU Admission to ICU Discharge	Hospital LOS	0.001	−1.380 ± 0.408 [−2.180–−0.580]
	ICU LOS	0.000	−0.542 ± 0.145 [−0.827–−0.257]
	Ventilator Days	0.011	−0.322 ± 0.126 [−0.569–−0.074]
	Mortality	0.148	0.009 ± 0.006 [−0.003–0.022]

The relationship between changes in creatinine level during two time frames (hospital admission to ICU admission, and ICU admission to ICU discharge) and various outcomes (hospital length of stay, ICU length of stay, ventilator days, and mortality). Change in Cr from Hospital to ICU admission was significantly correlated with ventilator days (*p* = 0.045). Change in Cr from ICU admission to ICU discharge was significantly correlated with hospital length of stay (*p* = 0.001), ICU length of stay (*p* = 0.000), and ventilator days (*p* = 0.011). Abbreviations: Cr = Creatinine; ICU = Intensive Care Unit; LOS = Length of Stay.

**Table 6:** Multivariable Correlation Between Changes in Creatinine Level and Hospital LOS, ICU LOS, and Ventilator Days.

Timeframe	Outcome	*p*-Value	Coefficient ± SE [95% CI]
Change in Cr Level from Hospital Admission to ICU Admission	Ventilator Days	0.090	−0.444 ± 0.262 [−0.959–0.070]
Change in Cr Level from ICU Admission to ICU Discharge	Hospital LOS	0.001	−1.394 ± 0.416 [−2.211–−0.577]
	ICU LOS	0.000	−0.543 ± 0.147 [−0.832–−0.255]
	Ventilator Days	0.014	−0.320 ± 0.130 [−0.575–−0.065]

Significant univariable correlations between changes in Cr level and hospital LOS, ICU LOS, and ventilatory days were scrutinized with multivariable analyses. Abbreviations: Cr = Creatinine; ICU = Intensive Care Unit; LOS = Length of Stay.

**Table 7:** Correlation of Cr Levels at All Time Points with Hospital LOS, ICU LOS, Ventilator Days, and Death, Stratified by Number of Sustained Neurologic Injuries.

Number of Injuries	Timeframe	Outcome	*p*-Value	Coefficient ± SE 95% CI
Overall	Cr at Admission	Hospital LOS	0.472	0.584 ± 0.811 [−1.008–2.175]
		ICU LOS	0.043	0.586 ± 0.288 [0.019–1.152]
		Ventilator Days	0.981	0.006 ± 0.250 [−0.486–0.497]
		Mortality	0.755	−0.004 ± 0.013 [−0.029–0.021]
	Cr at ICU Admission	Hospital LOS	0.135	1.289 ± 0.863 [−0.404–2.982]
		ICU LOS	0.001	1.029 ± 0.306 [0.429–1.630]
		Ventilator Days	0.031	0.576 ± 0.266 [0.054–1.098]
		Mortality	0.211	0.017 ± 0.013 [−0.010–0.043]
	Cr at ICU Discharge	Mortality	0.417	−0.005 ± 0.001 [−0.017–0.007]
1 Injury	Cr at Admission	Hospital LOS	0.174	0.617 ± 0.453 [−0.272–1.506]
		ICU LOS	0.061	0.404 ± 0.215 [−0.019–0.826]
		Ventilator Days	0.408	0.113 ± 0.136 [−0.155–0.391]
		Mortality	0.948	0.001 ± 0.011 [−0.022–0.023]
	Cr at ICU Admission	Hospital LOS	0.024	1.238 ± 0.162 [0.162–2.315]
		ICU LOS	0.003	0.766 ± 0.255 [0.255–1.276]
		Ventilator Days	0.002	0.508 ± 0.164 [0.186–0.831]
		Mortality	0.197	0.018 ± 0.014 [−0.009–0.045]
	Cr at ICU Discharge	Mortality	0.274	−0.017 ± 0.016 [−0.049–0.014]
2 Injuries	Cr at Admission	Hospital LOS	0.927	0.314 ± 3.433 [−6.437–7.065]
		ICU LOS	0.029	2.629 ± 1.196 [0.277–4.980]
		Ventilator Days	0.516	0.562 ± 0.865 [−1.139–2.263]
		Mortality	0.613	0.024 ± 0.048 [−0.070–0.119]
	Cr at ICU Admission	Hospital LOS	0.420	2.151 ± 2.662 [−3.083–7.385]
		ICU LOS	0.010	2.399 ± 0.929 [0.572–4.227]
		Ventilator Days	0.154	0.960 ± 0.672 [−0.362–2.285]
		Mortality	0.602	0.019 ± 0.037 [−0.053–0.093]
	Cr at ICU Discharge	Mortality	0.577	−0.004 ± 0.008 [−0.019–0.011]
3 Injuries	Cr at Admission	Hospital LOS	0.824	0.802 ± 3.591 [−6.337–7.942]
		ICU LOS	0.699	0.365 ± 0. 940 [−1.504–2.234]
		Ventilator Days	0.478	−1.111 ± 1.559 [−4.209–1.988]
		Mortality	0.420	−0.028 ± 0.035 [−0.098–0.041]
	Cr at ICU Admission	Hospital LOS	0.747	−0.938 ± 2.894 [−6.691–4.816]
		ICU LOS	0.936	0.061 ± 0.757 [−1.443–1.566]
		Ventilator Days	0.956	−0.070 ± 1.257 [−2.569–2.429]
		Mortality	0.958	−0.001 ± 0.028 [−0.058–0.055]
	Cr at ICU Discharge	Mortality	0.943	−0.002 ± 0.028 [−0.058–0.054]
4+ Injuries	Cr at Admission	Hospital LOS	0.469	10.642 ± 14.241 [−20.387–41.671]
		ICU LOS	0.159	7.644 ± 5.085 [−3.435–18.723]
		Ventilator Days	0.406	3.980 ± 4.622 [−6.090–14.050]
		Mortality	0.203	−0.478 ± 0.355 [−1.251–0.296]
	Cr at ICU Admission	Hospital LOS	0.549	−7.523 ± 12.209 [−34.124–19.078]
		ICU LOS	0.863	0.832 ± 4.712 [−9.435–11.098]
		Ventilator Days	0.924	0.397 ± 4.052 [−8.432–9.225]
		Mortality	0.198	0.411 ± 0.302 [−0.246–1.068]
	Cr at ICU Discharge	Mortality	0.133	−0.547 ± 0.339 [−1.285–0.192]

Overall, Cr at hospital admission was significantly associated with increased ICU LOS (*p* = 0.043), and Cr at ICU admission was significantly associated with increased ICU LOS (0.001) and increased number of vent days (*p* = 0.031). For patients who sustained TBI with no other injuries, Cr level at ICU admission was significantly associated with increased hospital LOS (*p* = 0.024), increased ICU LOS (*p* = 0.003), and increased number of vent days (*p* = 0.002). For patients who sustained TBI plus one other injury, Cr at hospital admission was significantly associated with increased ICU LOS (*p* = 0.029), and Cr at ICU admission was significantly associated with increased ICU LOS (*p* = 0.010). Abbreviations: Cr = Creatinine; ICU = Intensive Care Unit; LOS = Length of Stay.

**Table 8:** Multivariable Correlation of Cr Levels at All Time Points with Hospital LOS, ICU LOS, and Ventilatory Days, Stratified by Number of Sustained Neurologic Injuries.

	Timeframe	Outcome	*p*-Value	Coefficient ± SE [95% CI]
Overall	Cr at Admission	ICU LOS	0.038	0.621 ± 0.298 [0.036–1.206]
	Cr at ICU Admission	ICU LOS	0.002	1.010 ± 0.321 [0.379–1.640]
		Ventilator Days	0.062	0.529 ± 0.283 [−0.027–1.085]
1 Injury	Cr at ICU Admission	Hospital LOS	0.031	1.242 ± 0.575 [0.111–2.372]
		ICU LOS	0.003	0.817 ± 0.276 [0.273–1.360]
		Ventilator Days	0.003	0.538 ± 0.179 [0.186–0.889]
2 Injuries	Cr at Admission	ICU LOS	0.034	2.689 ± 1.262 [0.206–5.172]
	Cr at ICU Admission	ICU LOS	0.017	2.333 ± 0.973 [0.420–4.246]

After running multivariable regression to control for sex, age, race, ethnicity, and weight, all associations remained significant except for overall Cr at ICU admission and number of days requiring mechanical ventilation. Abbreviations: Cr = Creatinine; ICU = Intensive Care Unit; LOS = Length of Stay.

## Data Availability

The data was requested from the Elmhurst Trauma registry and extracted using electronic medical records after receiving approval from the Institutional Review Board at our facility (Elmhurst Hospital Center).
